# Dataset of serum proteomic spectra from tuberculosis patients detected by Raman spectroscopy and surface-enhanced Raman spectroscopy

**DOI:** 10.1016/j.dib.2019.104891

**Published:** 2019-11-26

**Authors:** Benjawan Kaewseekhao, Noppadon Nuntawong, Pitak Eiamchai, Sittiruk Roytrakul, Wipa Reechaipichitkul, Kiatichai Faksri

**Affiliations:** a Department of Microbiology, Faculty of Medicine, Khon Kaen University, Khon Kaen, Thailand; b Department of Medicine, Faculty of Medicine, Khon Kaen University, Khon Kaen, Thailand; c Research and Diagnostic Center for Emerging Infectious Diseases (RCEID), Faculty of Medicine, Khon Kaen University, Khon Kaen, Thailand; d National Electronics and Computer Technology Center (NECTEC), National Science and Technology Development Agency (NSTDA), Rama VI Rd., Pathumthani, Thailand; e National Center for Genetic Engineering and Biotechnology (BIOTEC), National Science and Technology Development Agency (NSTDA), Rama VI Rd., Pathumthani, Thailand

**Keywords:** Raman spectroscopy, Surface-enhanced Raman spectroscopy, Serum, Tuberculosis

## Abstract

In this data article, we present Raman spectroscopy (RS) and surface-enhanced Raman spectroscopy (SERS) data obtained using an InVia Reflex confocal Raman microscope (Renishaw; Wotton-under-Edge, UK) and processed using WiRE™ 4.2 software. The data include RS and SERS spectra detected, after removal of albumin, from the serum proteome of tuberculosis (TB) patient categories and controls (active tuberculosis; ATB, latent tuberculosis; LTBI, TB-exposed persons with undetected infection; EC, healthy controls; HC) using 532 nm and 785 nm laser wavelengths for RS and 785 nm for SERS. The RS and SERS data had high reproducibility (SERS; R^2^ = 0.988, RS at 785 nm; R^2^ = 0.972, RS at 532 nm; R^2^ = 0.9150). This data can be used for analysis of proteomic spectra based on RS and SERS for TB diagnosis and can also be compared to other populations. The spectral dataset based on normal, healthy control groups might be used as the control data for analysis of other diseases using RS and SERS approaches.

**Table:** Specifications Table

Subject	*Spectroscopy, Infectious Diseases*, Proteomics
Specific subject area	Raman Spectroscopy (RS) and surface-enhanced Raman spectroscopy (SERS) in serum protein samples detected among tuberculosis categories
Type of data	Raw and analysed data
How data were acquired	InVia Reflex confocal Raman microscope (Renishaw; Wotton-under-Edge, UK) and WiRE™ 4.2 software (Renishaw, UK) were used for data acquiring. In-house developed SERS chips (NECTEC, Thailand) were used for SERS analysis.
Data format	[.xlsx] and [.tiff]
Parameters for data collection	RS detected at 532 and 785 nm laser wavelengths and SERS detected at 785 nm laser wavelength.
Description of data collection	Protein fingerprint spectra based on RS and SERS were collected from serum proteome of 118 samples including 26 samples of active tuberculosis, 26 samples of latent tuberculosis infection, 34 samples of early clearance and 38 samples of healthy controls.
Data source location	Department of Microbiology, Faculty of Medicine, Khon Kaen University, Khon Kaen, Thailand
Data accessibility	Data is available with this publication

**Table:** 

**Value of the Data** • This dataset provide serum proteomic spectra from individuals with latent tuberculosis (TB) and those with active TB based on Raman Spectroscopy (RS) and SERS. This is the only serum proteomic spectral dataset of latent TB in a public database. • These data may be relevant for other researchers who (i) analyze the serum proteome based on RS and SERS, (ii) have a focus on TB diagnosis, especially for distinguishing between active and latent TB. • The dataset might be used for TB diagnostic applications by distinguishing among TB disease categories including active TB, latent TB, TB-exposed persons with undetected infection and healthy controls based on RS and SERS spectra. • The spectral dataset of the normal healthy control groups might be used as the control data for studies on other diseases based on Raman spectroscopy analysis.

## 1 Data

In this report, we present data of serum proteomic spectra detected among TB-infection categories using both Raman spectroscopy (RS) and surface-enhanced Raman spectroscopy (SERS). The TB-infection categories included unexposed individuals without infection (healthy controls; HC), exposed individuals without infection (early clearance; EC), those with latent infection (LTBI) and those with active TB disease (ATB). The protocol to acquire the dataset was approved by the Khon Kaen University Ethics Committee in Human Research (Ethics number HE611116).

**Fig. 1:**
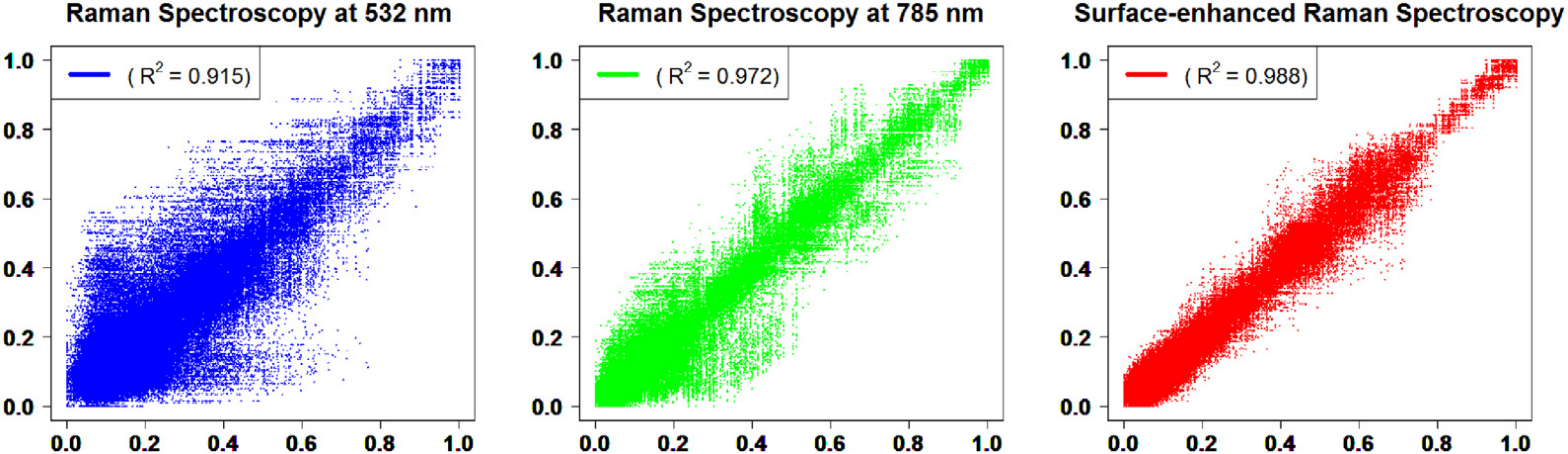
Reproducibility of Raman spectroscopy at 532 nm and 785 nm and SERS. A high correlation was found among sample replicates (average of 120 comparisons from 16 replicates) in Raman spectroscopy at 532 nm and 785 nm and SERS. The values at the upper left of each panel show average of R^2^ from each pair (all data are shown in Table 1).

The presented data include raw data of protein fingerprints detected among TB catagories (Supplementary Table S1). The high reproducibility of the data detected from both RS and SERS is shown in Fig. 1 and Table 1. The reproducibility of protein samples measured using SERS (R^2^ = 0.988) was slightly higher than that of RS at 532 nm (R^2^ = 0.915) and at 785 nm (R^2^ = 0.972) (Fig. 1 and Table 1).

**Table 1:** Reproducibility of Raman spectroscopy and SERS signal from 16 replicates from each sample. Correlation coefficient (R^2^) values of peak signal intensities in each pairwise comparison detected by Raman spectroscopy at 532 nm and 785 nm and surface-enhanced Raman spectroscopy are shown. Rep = Replications followed by the replicate number (ranging from 1 to 16).

Comparisons	RS		SERS	Comparisons	RS		SERS	Comparisons	RS		SERS
	532 nm	785 nm			532 nm	785 nm			532 nm	785 nm	
Rep1vs.Rep2	0.94	0.9	0.99	Rep5vs.Rep10	0.98	0.99	0.99	Rep3vs.Rep14	0.98	0.99	0.99
Rep1vs.Rep3	0.94	0.92	0.99	Rep6vs.Rep10	0.97	1	0.99	Rep4vs.Rep14	0.97	0.99	0.99
Rep2vs.Rep3	0.95	0.99	0.99	Rep7vs.Rep10	0.92	1	0.99	Rep5vs.Rep14	0.98	0.99	0.99
Rep1vs.Rep4	0.96	0.9	0.99	Rep8vs.Rep10	0.98	0.97	0.98	Rep6vs.Rep14	0.98	1	0.99
Rep2vs.Rep4	0.95	0.99	0.99	Rep9vs.Rep10	0.98	1	0.99	Rep7vs.Rep14	0.94	1	0.98
Rep3vs.Rep4	0.96	0.99	0.99	Rep1vs.Rep11	0.94	0.9	0.99	Rep8vs.Rep14	0.98	0.97	0.98
Rep1vs.Rep5	0.96	0.89	0.99	Rep2vs.Rep11	0.96	0.99	0.99	Rep9vs.Rep14	0.98	1	0.99
Rep2vs.Rep5	0.96	0.99	0.99	Rep3vs.Rep11	0.98	0.99	0.99	Rep10vs.Rep14	0.98	1	0.99
Rep3vs.Rep5	0.97	0.99	0.99	Rep4vs.Rep11	0.97	0.99	0.99	Rep11vs.Rep14	0.98	0.99	0.99
Rep4vs.Rep5	0.97	0.99	0.99	Rep5vs.Rep11	0.98	0.99	0.99	Rep12vs.Rep14	0.98	0.99	0.99
Rep1vs.Rep6	0.96	0.89	0.98	Rep6vs.Rep11	0.98	0.99	0.98	Rep13vs.Rep14	0.82	0.99	0.99
Rep2vs.Rep6	0.96	0.99	0.98	Rep7vs.Rep11	0.93	0.99	0.99	Rep1vs.Rep15	0.64	0.91	0.99
Rep3vs.Rep6	0.96	0.99	0.97	Rep8vs.Rep11	0.98	0.98	0.99	Rep2vs.Rep15	0.74	0.99	0.99
Rep4vs.Rep6	0.97	0.99	0.99	Rep9vs.Rep11	0.98	0.99	0.99	Rep3vs.Rep15	0.76	0.99	0.98
Rep5vs.Rep6	0.98	0.99	0.98	Rep10vs.Rep11	0.99	0.99	0.99	Rep4vs.Rep15	0.7	0.99	0.99
Rep1vs.Rep7	0.94	0.87	0.99	Rep1vs.Rep12	0.95	0.92	0.98	Rep5vs.Rep15	0.71	0.99	0.99
Rep2vs.Rep7	0.94	0.99	0.99	Rep2vs.Rep12	0.96	0.99	0.99	Rep6vs.Rep15	0.7	0.99	0.98
Rep3vs.Rep7	0.92	0.99	0.99	Rep3vs.Rep12	0.97	0.99	0.98	Rep7vs.Rep15	0.67	0.99	0.99
Rep4vs.Rep7	0.93	0.99	0.99	Rep4vs.Rep12	0.97	0.99	0.99	Rep8vs.Rep15	0.73	0.97	0.99
Rep5vs.Rep7	0.94	0.99	0.99	Rep5vs.Rep12	0.97	0.99	0.99	Rep9vs.Rep15	0.76	0.99	0.98
Rep6vs.Rep7	0.94	0.99	0.98	Rep6vs.Rep12	0.97	0.99	0.99	Rep10vs.Rep15	0.77	0.99	0.99
Rep1vs.Rep8	0.95	0.84	0.98	Rep7vs.Rep12	0.94	0.99	0.99	Rep11vs.Rep15	0.75	0.99	0.99
Rep2vs.Rep8	0.96	0.97	0.99	Rep8vs.Rep12	0.97	0.97	0.99	Rep12vs.Rep15	0.74	0.99	0.99
Rep3vs.Rep8	0.97	0.97	0.97	Rep9vs.Rep12	0.97	0.99	0.99	Rep13vs.Rep15	0.68	0.98	0.99
Rep4vs.Rep8	0.97	0.97	0.99	Rep10vs.Rep12	0.97	0.99	0.99	Rep14vs.Rep15	0.74	0.99	0.98
Rep5vs.Rep8	0.98	0.98	0.98	Rep11vs.Rep12	0.98	0.99	0.99	Rep1vs.Rep16	0.94	0.96	0.99
Rep6vs.Rep8	0.98	0.97	0.98	Rep1vs.Rep13	0.82	0.85	0.99	Rep2vs.Rep16	0.95	0.96	0.99
Rep7vs.Rep8	0.93	0.98	0.98	Rep2vs.Rep13	0.84	0.99	0.99	Rep3vs.Rep16	0.97	0.97	0.99
Rep1vs.Rep9	0.94	0.87	0.98	Rep3vs.Rep13	0.81	0.98	0.99	Rep4vs.Rep16	0.96	0.96	0.99
Rep2vs.Rep9	0.96	0.99	0.99	Rep4vs.Rep13	0.82	0.99	0.99	Rep5vs.Rep16	0.97	0.95	0.99
Rep3vs.Rep9	0.97	0.99	0.98	Rep5vs.Rep13	0.81	0.99	0.99	Rep6vs.Rep16	0.97	0.95	0.99
Rep4vs.Rep9	0.96	0.99	0.99	Rep6vs.Rep13	0.82	0.99	0.98	Rep7vs.Rep16	0.93	0.94	0.99
Rep5vs.Rep9	0.98	0.99	0.99	Rep7vs.Rep13	0.87	0.99	0.99	Rep8vs.Rep16	0.97	0.91	0.99
Rep6vs.Rep9	0.97	1	0.99	Rep8vs.Rep13	0.81	0.98	0.98	Rep9vs.Rep16	0.96	0.94	0.99
Rep7vs.Rep9	0.93	1	0.98	Rep9vs.Rep13	0.81	0.99	0.99	Rep10vs.Rep16	0.97	0.95	0.99
Rep8vs.Rep9	0.97	0.97	0.98	Rep10vs.Rep13	0.8	0.99	0.99	Rep11vs.Rep16	0.97	0.95	0.99
Rep1vs.Rep10	0.93	0.89	0.99	Rep11vs.Rep13	0.8	0.99	0.99	Rep12vs.Rep16	0.97	0.97	0.99
Rep2vs.Rep10	0.95	0.99	0.99	Rep12vs.Rep13	0.83	0.98	0.99	Rep13vs.Rep16	0.81	0.92	0.99
Rep3vs.Rep10	0.98	0.99	0.99	Rep1vs.Rep14	0.95	0.88	0.99	Rep14vs.Rep16	0.97	0.94	0.99
Rep4vs.Rep10	0.96	1	0.99	Rep2vs.Rep14	0.96	0.99	0.98	Rep15vs.Rep16	0.71	0.96	0.99

## 2 Experimental design, materials, and methods

### 2.1 Field collection of samples

In total, 118 serum samples including 26 samples of ATB, 26 samples of LTBI, 34 samples of EC and 38 samples of HC from the biobank of the Department of Microbiology, Faculty of Medicine, Khon Kaen University [1] were used. A diagnosis of ATB was based on clinical symptoms and positive evidence from a molecular test (Xpert MTB/RIF, Cepheid, Sunnyvale, CA, USA), acid-fast bacilli staining or bacterial culture. LTBI cases were defined based on a positive result of the QuantiFERON-TB Gold test (QFT) from healthy TB-exposed (persons having close contact with an ATB patient, such as working in TB wards for at least 6 months). The EC category was defined based on a negative result of the QFT in individuals having contact with ATB patients. Healthy controls (HC) were defined as apparently healthy persons with no evidence of TB exposure and having a negative result of the QFT.

### 2.2 Sample preparation

Albumin was excluded from each serum sample using protein filteration columns and centrifugation. Protein concentrations were measured using the Bradford protein assay and 0.8 μg of each protein sample were dropped onto aluminum foil. The samples were left to air dry for 3 minutes and detected using RS and SERS.

### 2.3 Protein fingerprint spectra collection and analysis

Raman Spectroscopy readings were taken on a InVia Reflex confocal Raman microscope (Renishaw; Wotton-under-Edge, UK) in a range of 179–1926 cm^−1^ for a 532 nm laser and 508–1632 cm^−1^ for a 785 nm laser. Data were generated based on RS detected at 532 and 785 nm laser wavelengths and SERS detected at 785 nm laser wavelength with 16 area points of detection in each sample. WiRE™ 4.2 software was used for data processing. To test the reproducibility of RS and SERS data, the average R^2^ score of 120 comparisons among 16 replications (detected from 1 to 16 area points) were calculated from 1010 peak positions in a sample from a randomly selected ATB case using the corrgram package in the R programming language. All SERS chips were developed by our group (NECTEC, Thailand). However, due to a shortage of SERS chips, we pooled the serum protein samples to match the available number of SERS chips.

## Funding

This work was supported by Invitation Research 2018, Faculty of Medicine (IN61210); and Research Fund for Supporting Lecturer to Admit High Potential Student to Study and Research on His Expert Program Year 2015 (581H223), Graduate Studies, Khon Kaen University.

## Ethics approval and consent to participate

The specimens from the biobank of the Department of Microbiology, Faculty of Medicine, Khon Kaen University. The protocol to obtain the dataset was approved by the Khon Kaen University Ethics Committee in Human Research (Ethic number HE611116).
